# Production of Conjoined Transgenic and Edited Barley and Wheat Plants for *Nud* Genes Using the CRISPR/SpCas9 System

**DOI:** 10.3389/fgene.2022.873850

**Published:** 2022-05-05

**Authors:** Yiming Zang, Qiang Gong, Yanhao Xu, Huiyun Liu, Hao Bai, Na Li, Lipu Du, Xingguo Ye, Caixia Lan, Ke Wang

**Affiliations:** ^1^ College of Plant Science & Technology, Huazhong Agricultural University, Wuhan, China; ^2^ Institute of Crop Science, Chinese Academy of Agricultural Sciences, Beijing, China; ^3^ Hubei Key Laboratory of Food Crop Germplasm and Genetic Improvement, Food Crops Institute, Hubei Academy of Agricultural Sciences, Wuhan, China

**Keywords:** nudum (nud) gene, genome editing, agrobacterium-mediated transformation, conjoined plants, naked grain

## Abstract

The *Nudum* (*Nud*) gene controls the caryopsis type of cereal crops by regulating lipid biosynthetic pathways. Based on the *HvNud* sequence and its homologous gene sequences in wheat, a conserved sgRNA was designed to obtain the mutants from the barley variety “Vlamingh” and the wheat variety “Fielder” *via Agrobacterium*-mediated transformation. A total of 19 and 118 transgenic plants were obtained, and 11 and 61 mutant plants were identified in T_0_ transgenic plants in barley and wheat after PCR-RE detection, and the editing efficiencies of the targeted gene were 57.9 and 51.7% in barley and wheat, respectively. The grain shape of the barley mutants was naked. Five different combinations of mutations for wheat *TaNud* genes were identified in the T_0_ generation, and their homozygous-edited plants were obtained in the T_1_ generation. Interestingly, the conjoined plants in which one plant has different genotypes were first identified. The different tillers in an individual T_0_ plant showed independent transgenic or mutant events in both barley and wheat, and the different genotypes can stably inherit into T_1_ generation, indicating that the T_0_ transgenic plants were the conjoined type. In addition, we did not find any off-target mutations in both barley and wheat. A candidate method for detecting putative-edited wheat plants was suggested to avoid losing mutations in this investigation. This study provides not only materials for studying the function of the *Nud* gene in barley and wheat but also a system for detecting the mutants in wheat.

## Introduction

Genome-editing technologies contain three types of sequence-specific nucleases (SSNs), viz., zinc-finger nucleases (ZFNs), transcription activator-like effector nucleases (TALENs), and clustered regularly interspaced short palindromic repeat-associated endonucleases (CRISPR/Cas) (Filippo et al., 2008; Lieber, 2010; Chapman et al., 2012; Jiang and Doudna, 2017; Mushtaq et al., 2019; Mushtaq et al., 2020; and Mushtaq et al., 2021a). At present, CRISPR/Cas is the most widely used genome-editing system in both animals and plants due to its easy assembling, straightforward guide RNA designing, and high activity (Mushtaq et al., 2021b). The most commonly used type for CRISPR/Cas is the type II system derived from *Streptococcus pyogenes* (SpCas9) that mainly recognizes the PAM (protospacer adjacent motif) sequence 5’-NGG-3’. To date, the genomes of many plants and crop species such as *Arabidopsis thaliana*, rice (*Oryza sativa*), tobacco (*Nicotiana tabacum* L.), tomato (*Lycopersicon esculentum*), maize (*Zea mays*), wheat (*Triticum aestivum* L.), barley (*Hordeum vulgare* L.), oilseed rape (*Brassica campestris* L), soybean (*Glycine max*), and chickpea (*Cicer arietinum* L.) have been edited using this technique (Li et al., 2013; Brooks et al., 2014; Zhang et al., 2016; Kelliher et al., 2017; Fiaz et al., 2019; Wu et al., 2020; Badhan et al., 2021; Fiaz et al., 2021).

Wheat and barley are two important cereal crops worldwide and are closely associated with social economic development, food production supply, food security, and human health and nutrition (Haas et al., 2019). Therefore, the improvement in the yield, quality, disease resistance, and stress tolerance of wheat and barley using CRISPR/SpCas9 technology is of significant value for the two crops (Abdelrahman et al., 2018). Recently, with the advancement of genetic transformation and the CRISPR/Cas system in wheat, several traits have been genetically modified by introducing mutations in the target genes using CRISPR/SpCas9. Wheat plants with mutations in *TaGW2* showed an increase in grain size (GS) and thousand-grain weight (TGW) (Wang et al., 2018; Zhang et al., 2018). The mutations in the wheat genes *TaMs2* led to the recovery of male sterility (Tang et al., 2021). Knocking-out wheat *TaPLA* or *TaMTL* genes induced haploid plant production (Liu et al., 2019a; Liu et al., 2020a). However, genome editing of barley is more straightforward than that of hexaploid wheat due to only one genome in the former. The editing case of barley *HvPM19* gene, which encodes an ABA-induced plasma membrane protein, was the first application of CRISPR/Cas9 in barley, and the mutants showed a dwarf phenotype (Lawrenson et al., 2015). Barley *Hvckx1* mutations led to reduced root growth and an increased number of tillers and grains (Gasparis et al., 2019). Mutations of *HvMORC1* induced with CRISPR/Cas9 resulted in plants with enhanced resistance against fungal pathogens in barley (Kumar et al., 2018).

Barley can be divided into two types based on the caryopsis: naked and hulled. Most domesticated barley cultivars have caryopses with adhering hulls that are known as hulled barley; some barley cultivars have a free-threshing feature and are called hulless (or naked) barley, especially the cultivars grown in the Tibetan Plateau of China. The caryopsis type in barley is controlled by the transcription factor gene *Nudum* (*Nud*), which encodes a protein in the ethylene response factor (ERF) family located on chromosome arm 7HL (Taketa et al., 2006). The barley *Nud* gene is homologous to the *Arabidopsis WIN1/SHN1* transcription factor gene, which is thought to function in lipid biosynthesis. It has been shown that the caryopsis surface in hulled barley is overlaid with lipid compounds, which penetrate to the inner side of the hull to form the adhesion organ (Taketa et al., 2008).

Studies on X-ray-induced naked mutation alleles (Taketa et al., 2008) and *Nud* locus re-sequencing in 162 barley cultivars (Yu et al., 2016) showed that amino acid substitutions and frame shifts in *Nud* led to loss-of-function and further resulted in the naked phenotype. Moreover, a single nucleotide polymorphism of *HvNud* (T643A), which generated an amino acid substitution of valine (Val, V) by aspartate (Asp, D) at position 148 (Val 148 Asp), could lead to the naked caryopsis type in barley (Yu et al., 2016). There are also three homologous genes for the *Nud* gene in wheat that are located on chromosomes 7AL, 7BL, and 7DL, but their functions are presently unknown. Additionally, *Nud* may also influence other traits in barley. The role of the *Nud* gene has not been fully investigated at present, especially its roles in controlling the naked hull phenotype in barley for efficient breeding of naked barley varieties. Therefore, it is necessary to characterize the function of *Nud* genes in detail in wheat and barley.

In this study, mutants in *HvNud* and *TaNud* were generated by CRISPR/SpCas9 in both barley and wheat. The efficiency and heritability of the mutations were investigated in wheat. Moreover, this is the first report in which we found that different tillers in an individual T_0_ plant were independent transgenic or genome-editing events in both barley and wheat, called as conjoined plants. Conjoined plants mean that an individual plant contains different genotyping which is produced possibly from different contiguously transformed cells and developed like one transgenic plant/event. Additionally, potential off-target sites for *HvNud* and *TaNud* in barley and wheat, respectively, were detected and no off-target mutations were found. A method to test candidate-edited wheat plants was put forward to avoid losing mutations in this study. In the present study, the *Nud* gene mutants developed in barley and wheat will be valuable for further investigation of the functions of this gene in the two crops.

## Materials and Methods

### Plant Materials

The barley cultivar ‘Vlamingh’ and the wheat cultivar ‘Fielder’ were kindly provided by the National Crop Germplasm Bank, Institute of Crop Sciences, Chinese Academy of Agricultural Sciences, Beijing, China. The plants of both species were cultured in an environmental growth chamber at 24°C−16 h light/18°C−8 h dark with a light intensity of 300 μmol m^−2^ s^−1^ at 45% relative humidity.

### Designing the sgRNA for the *Nud* Genes

The sequence of the barley *HvNud* gene that determines hulled vs. naked caryopsis was obtained from NCBI (https://www.ncbi.nlm.nih.gov/, Gene Bank accession AP009567.1). The wheat *Nud* genes (*TaNud*) were identified using *HvNud* as a query in a BLAST search of the IWGSCv1 wheat genome (https://urgi.versailles.inra.fr/blast/blast.php). In order to simultaneously edit the *Nud* genes in barley and wheat, a conserved 20-bp sgRNA sequence (5’-CGG​CTC​CTT​GTT​GAG​CTC​GA-3’) containing a *SacI* restriction site was selected as the target site for both *HvNud* and *TaNud*. Off-target sites related to the 20-bp sgRNA sequence were predicted by searching the target sequence in the IBSCv2 barley genome (https://webblast.ipk-gatersleben.de/barley_ibsc/) and the IWGSCv1 wheat genome.

### Vector Construction

The full DNA sequence encoding SpCas9 (Ma et al., 2015) was inserted into the expression vector pWMB110 to generate a new plasmid, pWMB110-SpCas9 (Liu et al., 2019a). The wheat *TaU3* promoter was cloned onto plasmid pUC18 as a template, and the sgRNA designed for the *Nud* gene was linked with the *TaU3* cassette by overlapping PCR (Ma et al., 2015). The *TaU3* promoter–sgRNA expression cassette was then amplified and inserted onto the vector pWMB110-SpCas9 at the *MluI* cloning site to generate the recombinant plasmid pWMB110-SpCas9-Nud (Supplementary Figure S3). The final vector pWMB110-SpCas9-Nud was transferred into *Agrobacterium* strain C58C1 for transformation of wheat and barley.

### *Agrobacterium*-Mediated Plant Transformation

Immature barley and wheat grains were collected approximately 14 days post anthesis (DPA). The immature grains were sterilized with 75% ethanol for 1 min, followed by 5% sodium hypochlorite for 15 min, and washed five times with sterile water.

Fresh immature embryos of wheat were isolated and transformed by *Agrobacterium*-mediated transformation to generate transgenic plants following the protocol described by Ishida et al. (2015) with slight modifications. In brief, immature wheat embryos were incubated with *Agrobacterium* strain C58C1 harboring the vector for 5 min in a WLS-inf medium at room temperature and co-cultivated for 2 days on the WLS-AS medium with the scutellum facing upward at 25°C in darkness. After co-cultivation, embryonic axes were removed with a scalpel, and the scutella were transferred onto plates containing the WLS-Res medium for delay culture for 5 days under the same conditions. Afterward, tissues were cultured on the WLS-P5 medium with 5 mg L^−1^ phosphinothricin (PPT, Sigma, 45,520) for callus induction. After two weeks, the calli were placed on the WLS-P10 medium with 5 mg L^−1^ PPT for 3 weeks in darkness. The 1/2 MS medium containing 5 mg L^−1^ PPT without zeatin was used for differentiation of embryonic calli other than the LSZ-P5 medium in the methods of Ishida et al. (2015) at 25°C with 100 μmol m^−2^ s^−1^ light. Regenerated shoots were transferred into cups filled with 1/2 MS medium with 5 mg L^−1^ PPT for shoot elongation and root formation.

Barley transformation was performed following the previously published protocols with a slight modification (Bartlett et al., 2008). Immature embryos of barley were isolated after sterilization of the immature grains by the same methods as wheat, subsequently incubated with *Agrobacterium* for 10 min, and co-cultivated for 2 days on CM medium. Then, embryo axes were remove, and the scutella were cultured on the first selection medium with 5 mg L^−1^ PPT. After 2 weeks, tissues were transferred onto the second selection medium with 10 mg L^−1^ PPT. After three weeks, embryonic calli were cultured on the DM medium with 5 mg L^−1^ PPT at 25°C with 100 μmol m^−2^ s^−1^ light for differentiation. Shoots were timely moved into a plastic box containing the RT medium. Last, plants were transplanted into pots filled with soil.

### Detection of Transgenic Plants and Edited Mutations

Mixed leaf samples from different tillers of T_0_ transgenic plants at the jointing stage were collected for genomic DNA extraction using the CWBIO NuClean Plant Genomic DNA Kit (CWBIO Biotech Co., Ltd.). The *SpCas9* and target genes *HvNud* and *TaNud* in the T_0_ transgenic plants were amplified with gene-specific primers (Supplementary Table S1) using 2X Taq Master Mix (Vazyme Biotech Co., Ltd.) for positive detection. Two types of primers were employed to amplify the *TaNud* gene: 1) gene-conserved primer pairs designed by the barley *HvNud* gene to simultaneously amplify the homologous wheat *TaNud* genes from the A, B, and D genomes and 2) gene-specific primer pairs designed to amplify the individual wheat gene from each of the three genomes (Supplementary Table S1).

Mutations in the *Nud* genes were detected using a polymerase chain reaction-restriction enzyme (PCR-RE) approach. For this test, the amplification reactions were performed in a volume of 20 μl consisting of 2× PCR Mix, 50 ng of genomic DNA, and 0.25 μM of each primer. PCR amplification was performed in a Veriti 96 PCR system (Applied Biosystems) using the following program: an initial denaturation step at 94°C for 5 min, followed by 34 cycles of 94°C for 1 min, 60°C for 45 s and 72°C for 1 min and a final elongation step at 10 min at 72°C. The restriction enzyme digestion of the PCR products was performed in a 20 μl reaction volume containing the appropriate restriction enzyme buffer and 0.2 μl *Sac*I enzyme for 4–6 h at 37°C. The digested products were separated in a 1.5% agarose gel and visualized using a GelDoc XR System (Bio-Rad, United States). To distinguish the different mutant types, the biggest band in the PCR-RE test was directly sequenced for homozygous mutations or indirectly sequenced after cloning into the pMD18-T vector for heterozygous mutations (TaKaRa, Dalian, China) at Sangon Biotech (Shanghai, China). The software BioEdit is used for sequence alignment and analysis (Hall, 2011). The mutations were identified by aligning the sequenced sequences with the referenced sequences of the targeted genes.

## Results

### Analysis of the *HvNud* Gene in Barley and its Homologs in Wheat

**FIGURE 1:**
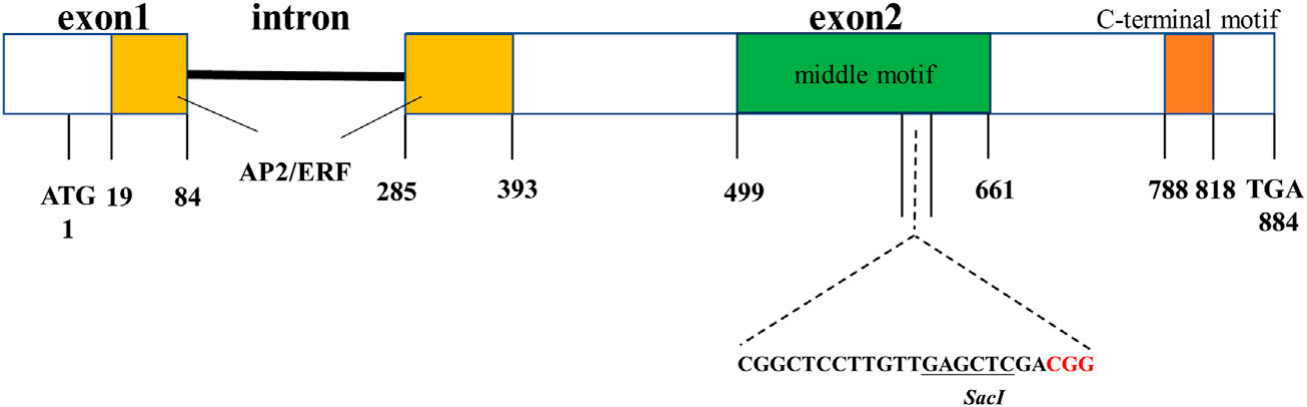
Structure diagram of the *HvNud* gene. Boxes indicate exons, and the black bar between the boxes indicates intron.

The structure of the barley *HvNud* gene (*HORVU7Hr1G089930*) consists of two exons and one intron, and its ORF encodes a deduced protein of 227 aa (Taketa et al., 2008, Figure 1). By using *HvNud* as a query in BLAST searches of the wheat genome database, three orthologous *TaNud* genes (*TraesCS7A02G376300*, *TraesCS7B02G277800*, and *TraesCS7D02G372700*) located on chromosomes 7A, 7B, and 7D were found. The *TaNud* genes have the same gene structure as *HvNud*. All of the proteins predicted from the *Nud* gene sequences contain an AP2/ERF domain, a middle motif, and a C-terminal motif (Figure 1). Moreover, the DNA sequence similarity between the *HvNud* and *TaNud* genes is 87.7%, and their similarity in protein sequence is as high as 94%. A conserved sequence (5’-CGG​CTC​CTT​GTT​GAG​CTC​GA-3’, containing a *SacI* restriction site) located in the second exon of both *HvNud* and *TaNud* was selected as the sgRNA for editing.

### Detection of *HvNud* Gene-Edited Mutations in T_0_ Transgenic Barley Plants

**FIGURE 2:**
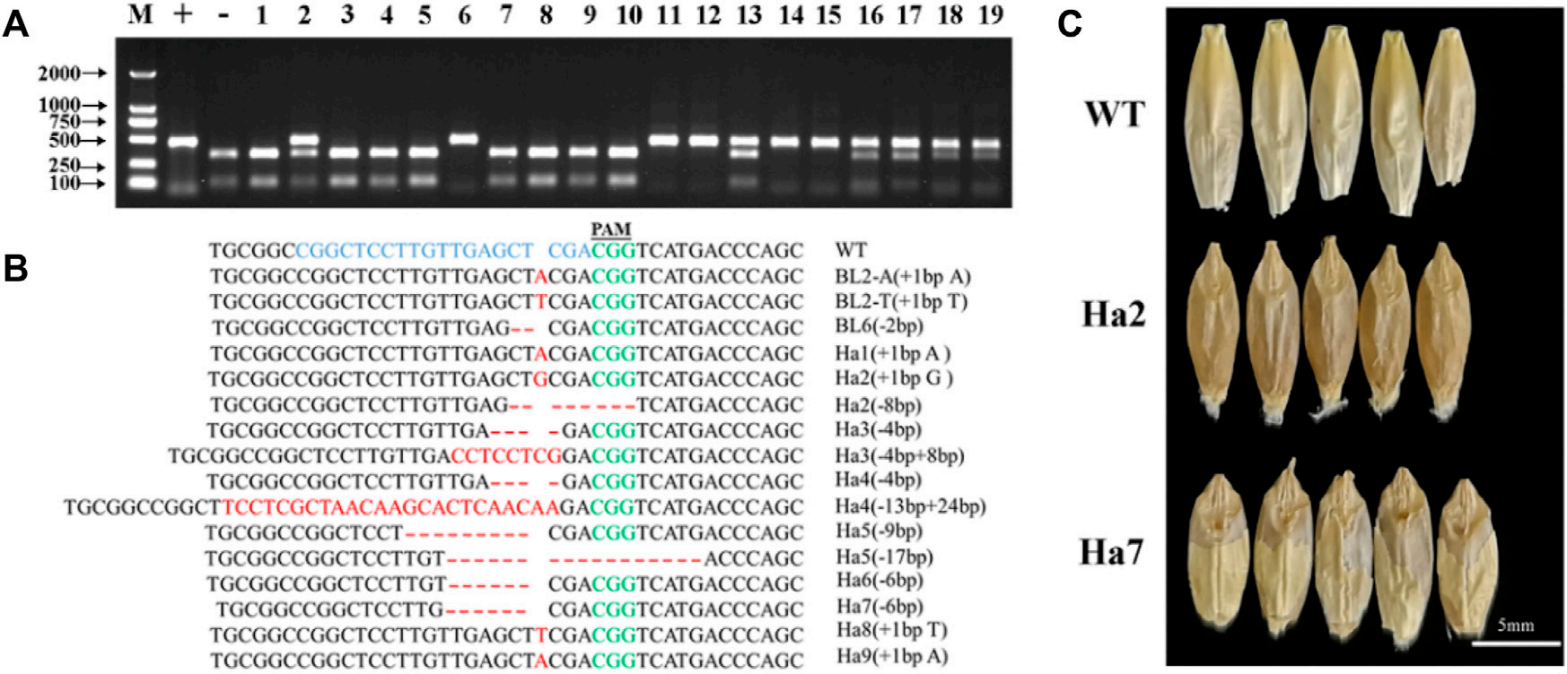
Detection and phenotyping of *HvNud* mutations in transgenic barley plants. **(A)** PCR products of *HvNud* digested with *Sac*I restriction enzyme, M: DNA marker; +: positive control; −: negative control; 1–10: B1-BL10; 11–19: Ha1–Ha9. **(B)** InDel mutations in the *HvNud* gene in edited T_0_ transgenic barley plants. Nucleotide bases shown in red differ from the target sequence in the sgRNA. **(C)** Phenotype of T_1_ grains harvested from mutant plants in comparison to WT grain and T_1_ grains collected from *HvNud* knockout T_0_ mutants. WT: wild type without threshing; Ha2: frame-shift mutation after threshing; Ha7: 6 bp deletion mutation after threshing.

In total, 19 T_0_ transgenic barley plants (BL1 to BL10 and Ha1 to Ha9) were generated from two experiments by *Agrobacterium*-mediated transformation using immature embryos. A pair of primers, HvNud-330F and HvNud-816R, was used to amplify the *HvNud* gene from mixed leaves in an individual plant, and the PCR products were then digested with *SacI*. Three types of band patterns (Figure 2A) were found in the PCR-RE experiment: heterozygous monoallelic mutants gave three bands, biallelic mutants only gave a band of 487 bp, and non-mutants as well as wild-type (WT) plants gave completely digested bands of 359 bp and 128 bp. A total of 11 mutant plants were obtained (Figure 2A), and the editing efficiency in the T_0_ transgenic barley plants was 57.9%. A total of five and six mutant plants were confirmed to be biallelic and heterozygous monoallelic mutants in the experiment, respectively (Supplementary Table S2).

**FIGURE 3:**
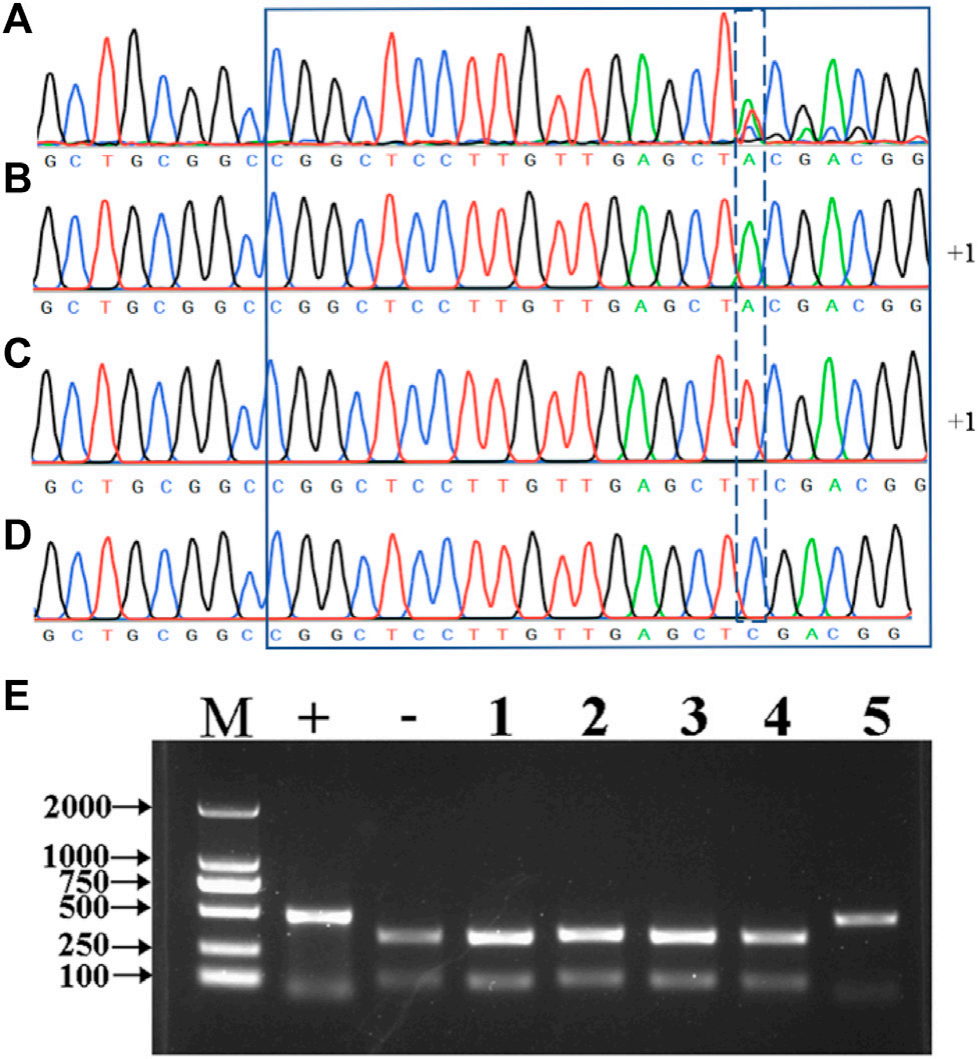
**(A)** Sequencing of the edited sites of the *HvNud* gene using the PCR products of BL2. **(B–D)** Sequencing by using the subclonings of BL2 PCR products on the vector. **(E)** Detection of *HvNud*-edited mutations in different tillers of Ha3 in T_0_ generation by PCR-RE. M: DNA marker; +: positive control; -: negative control; 1–5: five different tillers of Ha3.

The undigested PCR products from the mutant plants were directly sequenced, and the results showed that the 1-bp insertion mutation type appeared in the five mutant plants, while there are nine different mutation types in T_0_ generation (Figure 2B, Supplementary Table S2). It is interesting to find three peaks after the 5’-CGG​CTC​CTT​GTT​GAG​CT-3’ target sequence in BL2 (Figure 3A), which suggests that there might be three types of the *HvNud* sequence at this nucleotide position. The PCR-amplified DNA fragments from the targeted sequence in BL2 were subcloned onto vector pMD18-T and sequenced. Surprisingly, the sequencing results showed that there were three types of *HvNud* sequence at the target site in BL2, which confirmed the interpretation of the results: the first type has an A nucleotide insertion (Figures 2B, 3B); the second type has a T nucleotide insertion (Figures 2B, 3C); and the third type remains unchanged from the WT (Figure 3D). Unfortunately, we did not harvest seeds from plant BL2. The other mutant plants were randomly selected for mutation detection by tillers. Also, the similar results to BL2 were found in Ha3, in which only one tiller (Ha3-5) was a biallelic mutant and the other four tillers had no mutations after PCR-RE detection (Figure 3E). Sequencing results showed that one DNA strand in tiller Ha3-5 had a 4 bp deletion and the other DNA strand had a 4 bp deletion and an 8 bp insertion (−4 bp +8 bp) (Figure 2B). These results proved that the different tillers in Ha3 were different independent events.

### Inheritance of *HvNud* Mutation Site in T_1_ Generation

The T_0_ transgenic barley plants numbered Ha2, Ha3, Ha4, Ha6, and Ha7 produced seeds normally. The seeds of Ha2 and Ha4 were naked (Figure 2C). But, the seeds of Ha6 and Ha7 were hulled and the same as their WT. In addition, the lemma covering the seeds was difficult to be removed due to 6 bp deletion in the two mutants (Figures 2B,C). As different tillers in Ha3 belonged to different genotypes (Figure 3E), the seeds in this plant were harvested by tillers. We found that the seeds from Ha3–5 were naked, and the seeds from other tillers were hulled. Moreover, the results by PCR-RE revealed that all the T_1_ plants from Ha3–5 were biallelic mutants, while no mutations were found in the descendants of the other four tillers of Ha3 (Ha3‐1, Ha3‐2, Ha3‐3, and Ha3‐4) (Supplementary Figure S1). Sequencing results confirmed that Ha3‐5‐3 was a homozygous mutant with 4 bp deletion, while Ha3‐5‐1 and Ha3‐5‐2 were heterozygous biallelic mutants with −4 bp/−4 bp +8 bp. These findings were consistent with the detection results of Ha3 in T_0_ generation (Figure 2B). The aforementioned results confirmed that the different tillers of Ha3 can inherit stably following the Mendelian rule.

### Detection of Mutations in Different Tillers in T_0_ Transgenic Wheat Plants

**FIGURE 4:**
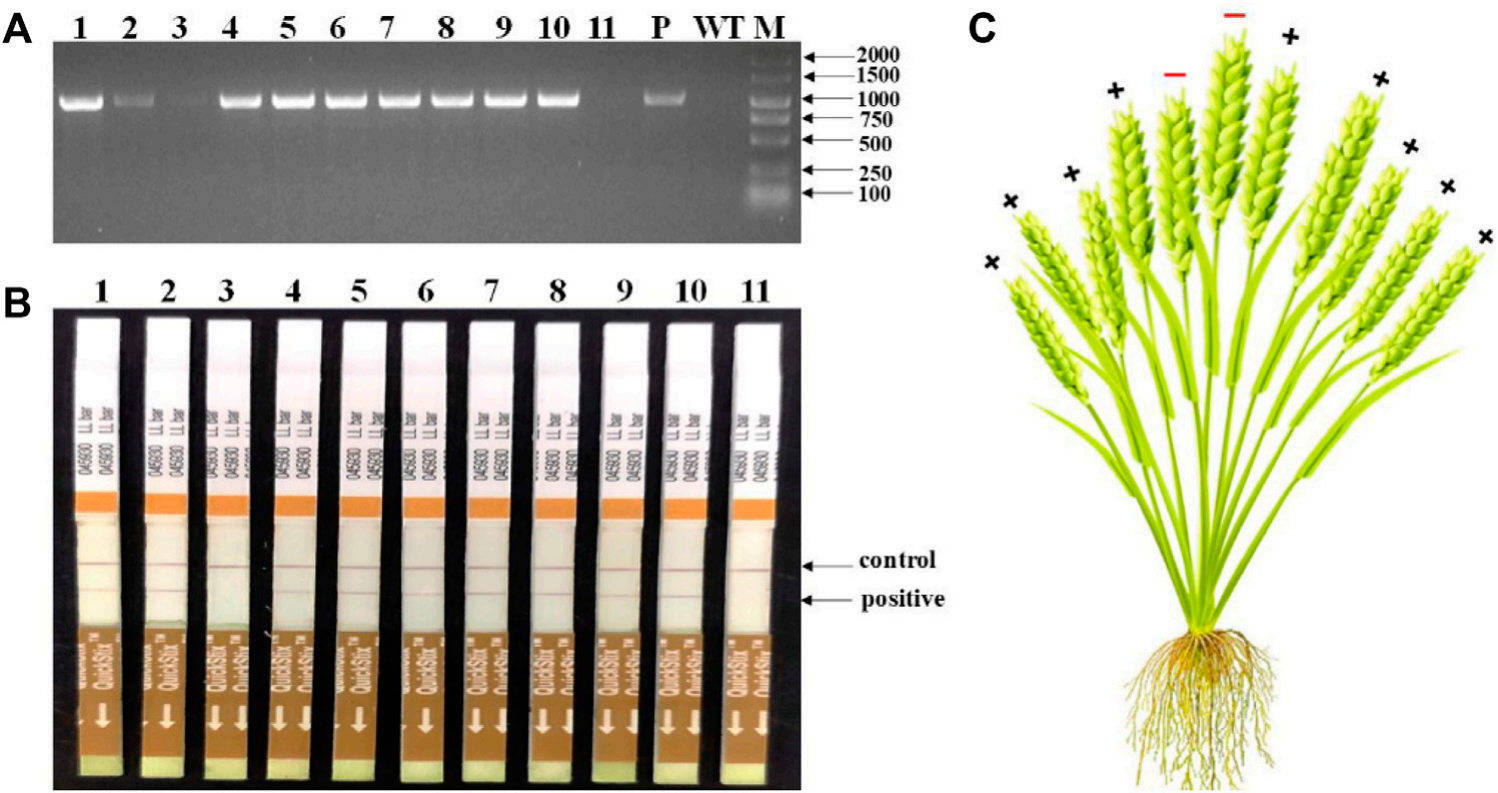
Detection of *SpCas9* and *bar* genes in different tillers of WL8 by PCR and QuickStix strips, respectively. **(A)** Detection of *SpCas9* gene by PCR. 1–11: different tillers of WL8; WT: wild type by PCR-RE; P: PCR products without digestion; M: DNA marker. **(B)** Detection of Bar protein by QuickStix strips. **(C)** Schematic diagram of conjoined transgenic wheat plant WL8. +: positive tiller; −: negative tiller.

**FIGURE 5:**
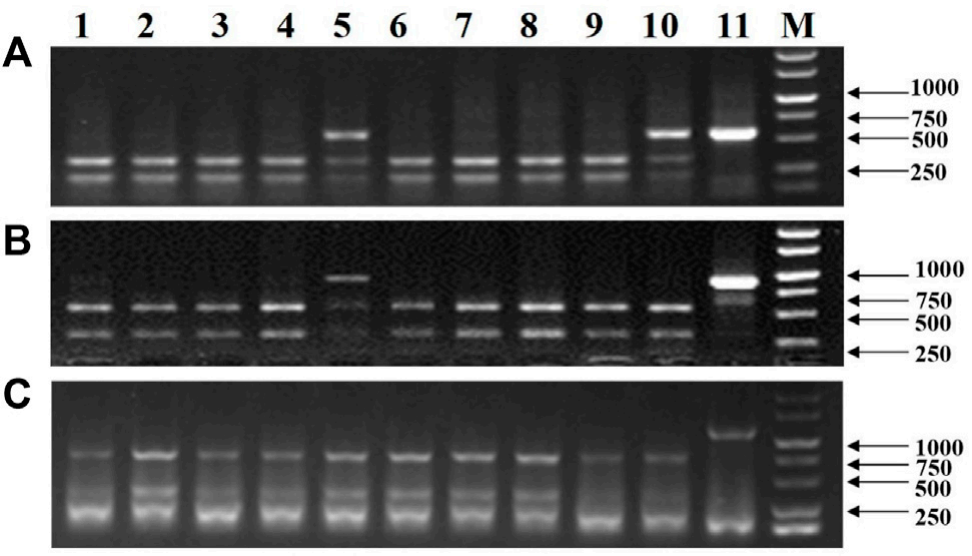
Detection of mutations of the wheat *TaNud* genes in different tillers of WL15 by PCR-RE. **(A)** *TaNud*-*7A*. **(B)** *TaNud*-*7B*. **(C)** *TaNud*-*7D*. 1–10: different tillers of WL15; 11: positive control; M: DNA marker.

Totally, 118 transgenic wheat plants were generated and named from WL1 to WL118. At the outset of the testing, five individual T_0_ plants (WL1, 5, 8, 15, and 16) were randomly selected to test the *bar* gene using QuickStix strips and *SpCas9* gene by PCR in different tillers. The results showed that all the tillers from plants WL1, 5, 15, and 16 were positive, while two of 11 tillers on plant WL8 were negative for the *SpCas9* (Figure 4A) and *bar* genes (Figure 4B). Moreover, both the negative and positive events from the tillers can be steadily detected in T_1_ generation. The mutations in the *TaNud* genes were further detected in the four individual plants using the PCR-RE assay and positive detections for *SpCas9* and *bar* genes, and edited mutations for the *TaNud* gene were identified in plants WL5 and WL15. In particular, one tiller showed mutations in genomes A and B, another tiller showed mutations in genome A, and the other eight tillers showed no mutations (Figure 5) in plant WL15. These results were consistent with the findings achieved in plant Ha3 in barley, indicating that different tillers in an individual T_0_ plant might belong to different independent transgenic or edited events.

### Mutation Frequency and Type of *TaNud* Genes in T_0_ Transgenic Wheat Plants

**FIGURE 6:**
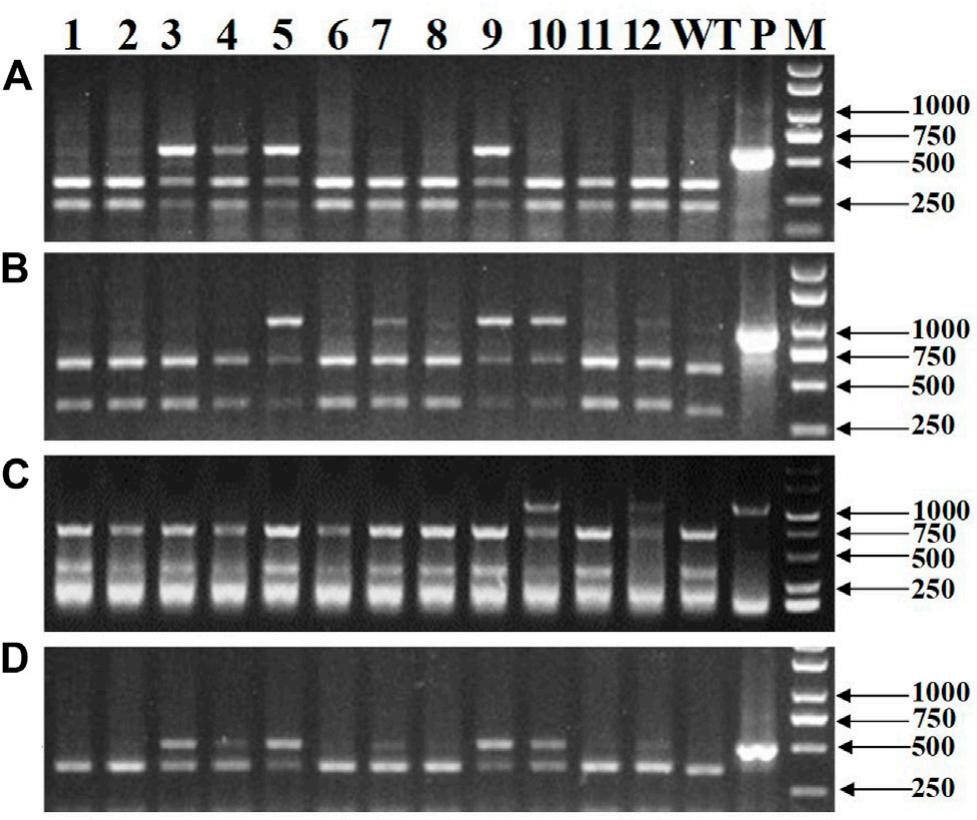
Detection of mutations of wheat *TaNud* genes by PCR-RE assay using specific primers and the conservative primers. **(A)** *TaNud*-*7A*-specific primers. **(B)** *TaNud*-*7B*-specific primers. **(C)** *TaNud*-*7D*-specific primers. **(D)** Conservative primers. 1: WL1; 2: WL2; 3: WL4; 4: WL5; 5: WL11; 6: WL12; 7: WL18; 9: WL46; 10: WL50; 11: WL51; 12: WL56; WT: wild type by PCR-RE; P: PCR products without digestion; M: DNA marker.

Genomic DNA was extracted from the mixed leaf samples of the T_0_ transgenic plants and detected for mutations in the three *TaNud* genes on chromosomes 7A, 7B, and 7D by PCR-RE assay using the gene-specific primers (Figures 6A–C). Mutations with the *TaNud* genes were detected in 61 T_0_ transgenic plants (Table 1), and the total editing efficiency of the target genes was 51.7%. In detail, the editing efficiencies for the three *TaNud* genes on chromosomes 7A, 7B, and 7D were 24.6, 33.1, and 8.5%, respectively. The efficiency for the simultaneous mutation of any two genes in a single plant was 14.4% (Table 1), and there was no plant identified with simultaneous mutations in the three *TaNud* genes. Theoretically, there were seven combinations (*aaBBDD*, *AAbbDD*, *AABBdd*, *aabbDD*, *AAbbdd*, *aaBBdd* ***,*** and *aabbdd*) of biallelic mutations in the three genes in the edited T_0_ wheat plants, but only five biallelic mutation types were obtained in this study (Table 1).

**TABLE 1:** Summary of the mutations of the *TaNud* genes in T_0_-edited wheat plants.

Mutation allele	Mutant plant	Mutation rate (%)	Plant ID
7A	17	14.4	WL−4, 5, 10, 14, 20, 40, 48, 52, 75, 89, 92, 105, 106, 107, 110, 114, and 117
7B	22	18.6	WL−18, 21, 23, 32, 33, 36, 43, 53, 57, 59, 62, 63, 68, 76, 77, 80, 84, 90, 94, 96, 97, and 118
7D	5	4.2	WL−56, 67, 69, 95, and 109
7A and 7B	12	10.2	WL−11, 15, 17, 24, 27, 38, 46, 66, 74, 104, 113, and 115
7B and 7D	5	4.2	WL−50, 60, 82, 91, and 101

Lower case letters represent mutant types; capital letters represent wild type.

### Application Comparison of the Gene-Conserved and -Specific Primers Used to Detect Edited Plants

A total of nineteen T_0_ transgenic wheat plants which carried the five combinations of biallelic mutations in the three *TaNud* genes were used to compare the gene-conserved primers (HvNud-330F and HvNud-816R) and gene-specific primers for the three alleles by PCR-RE assay. The results using the conserved primers (Figure 6D) showed the mutated DNA fragments as long as there was a mutation in any one of the three genes on chromosomes 7A, 7B, and 7D (Figures 6A–C). From this result, we can infer that the conserved primers are able to detect all of the mutations in the three different *TaNud* genes. Therefore, the conserved primers can be used to quickly screen the transgenic plants, and a detailed confirmation of the mutation types can then be performed using the specific primers from the plants which contained mutations. This approach is necessary to be adapted when large groups of transgenic plants are obtained, or the editing efficiency in an experiment is low.

### Inheritance of *TaNud* Mutation Sites in T_1_-Edited Wheat Plants

**FIGURE 7:**
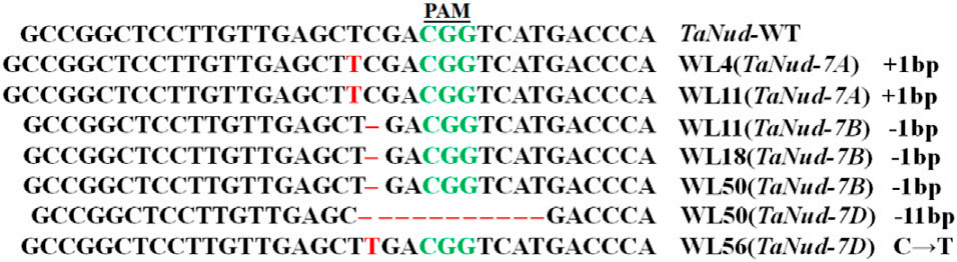
InDel mutations in *TaNud* genes in the edited T_1_ transgenic wheat plants.

Genetic segregation of the mutations in the *TaNud* genes was detected in the T_1_ generation by PCR-RE using gene-specific primers and DNA sequencing. For this purpose, five T_0_ plants (WL4, WL11, WL18, WL50, and WL56), in which each plant belonged to a different mutation-type category, were selected for further characterization in the next generation. A total of ten T_1_ plants were detected in each of the edited lines. The frequencies of homozygous mutants were 30, 40, and 40% for the edited types *aaBBDD*, *AAbbDD*, and *AABBdd*, respectively (Table 2). The frequencies of simultaneous homozygous mutation plants at two loci were 20 and 10% for *aabbDD* and *aabbDD*, respectively (Table 2). The segregation ratios of the edited sites in the T_1_ generation plants did not follow the Mendelian heritance pattern, which might be either due to the T_1_ population being small or their T_0_ generations being conjoined. In total, five types of homozygous mutations were identified in the T_1_ plants, and DNA sequencing showed that the mutations in the *TaNud* genes included small nucleotide insertions, deletions, and substitutions (Figure 7, Supplementary Table S2).

**TABLE 2:** Summary of mutations in the *TaNud* genes in T_1_-edited wheat plants.

T_0_ line	T_0_-edited allele	T_1_-edited type		
		Homozygous	Heterozygous	WT
WL4	aaBBDD	3	3	4
WL18	AAbbDD	4	5	1
WL56	AABBdd	4	3	3
WL11	aabbDD	2	8	0
WL50	AAbbdd	1	9	0

### Detection of Off-Target Mutations

The presence of potential off-target mutated sites in barley and wheat was further analyzed. Two putative off-target sites which had three or five SNPs compared with the target sequences were predicted for each of *HvNud* and *TaNud* genes from the IBSCv2 barley genome and IWGSCv1 wheat genome by a BLAST search (Supplementary Table S3). Specific PCR primers were designed to amplify the potential off-target regions (Supplementary Table S3) in two T_0_ barley transgenic plants and 61 T_0_ wheat transgenic plants. The PCR-RE results verified that there were no off-target mutations happened in our present experiments. Therefore, the CRISPR/Cas9 system precisely targeted the selected sites in the *Nud* gene sequences of the two crops in this study.

## Discussion

Compared to other techniques used to induce mutations, such as ethyl methyl sulfonate (EMS) treatment and ray irradiation, the CRISPR/Cas9 system is much precise and efficient in generating specific mutations. Therefore, CRISPR/Cas9 has been the common choice to induce mutations for target gene function analysis and crop improvement because the genetic transformation efficiency of wheat and barley has been significantly improved (Bartlett et al., 2008; Wang et al., 2016); the application of CRISPR/Cas9 will also be more widespread in these two important crops. However, since wheat is a hexaploid plant, most wheat genes are present in at least three copies, and the difficulty of editing multiple target genes simultaneously may limit the application of CRISPR/Cas9 in wheat.

### Mutant Induction of the *Nud* Gene by CRISPR/Cas9 in Barley and Wheat

In this study, five biallelic mutant plants and six monoallelic heterozygous mutant plants were obtained for editing the barley *HvNud* gene with an editing efficiency of 57.9%. Biallelic mutation efficiency was up to 26.3%, and the naked grain phenotype was observed in three T_1_ lines Ha2, Ha3, and Ha4. Wheat has always lagged behind other cereal crops with respect to the applications of genetic modification technologies due to its complex polyploid genome. Although there were many studies on the use of CRISPR/Cas9 in wheat, there were few reports describing the simultaneous mutation of target genes located on A, B, and D genomes (Wang et al., 2020). With the optimization on vector construction and the improvement on editing efficiency by the CRISPR/Cas9 system for wheat, the application of genome-editing technology will soon become routine in wheat. By using the optimized CRISPR/Cas9 system in wheat (Liu et al., 2019a), the editing efficiency of *TaWaxy* and *TaMTL* genes in T_0_ plants reached 80.5 and 57.5%, respectively. In our current study, the editing efficiency of the three *TaNud* alleles also reached 51.7%, but the editing efficiency of *TaNud*-7*D* was only 8.5%. The low editing efficiency of *TaNud*-7*D* resulted in failure to obtain plants carrying mutations in all three *TaNud* genes on the three wheat genomes. Therefore, the same target site might lead to different editing efficiencies for different homoeologous genes. It is also possible that the editing efficiency for genome D is lower than that for genomes A and B due to the special structure in the target region of genome D in wheat. In a word, CRISPR/Cas9 can be employed to accurately generate targeted mutations for crop improvement.

### Potential Function of the *HvNud* Mutants by CRISPR/Cas9

Generally, the hulled barley is used for distilling, brewing, and animal feed, and the naked barley is an important food source in Asia and northern Africa. Compared with the hulled barley, the naked barley is easy to separate from its outer glume, which is convenient for processing and eating. Moreover, the naked barley has a high protein level, high β-glucan, some rare nutrients, and trace element contents (Östman et al., 2006). The barley β-glucan can reduce blood LDL-cholesterol and visceral fat obesity (Tiwari and Cummins, 2011; Aoe et al., 2017). Therefore, the mutants of *HvNud* gene could not only convert the hulled germplasm into naked barley for dietary purposes but also increase the nutrition value in barley grains.

### Identification of Conjoined T_0_ Transgenic Barley and Wheat Plants

In general, an individual T_0_ transgenic plant has been thought to represent a single transgenic event. Previously, the transgenic seedlings arising from the same embryo were even considered to represent the same transgenic event. In a previous study, different phenotypes for editing the wheat *TaQ* gene were observed in different tillers of a single transgenic plant (Liu et al., 2020b). In our present study, different independent transgenic events were first identified in different tillers of an individual plant and could inherit into next generations in barley and wheat. In T_0_ barley plants, Ha3 displayed two different genotypes in different tillers (Figure 3E), and the two genotypes could stably inherit in T_1_ generation, respectively (Supplementary Figure S1). Theoretically, all of the transgenic plants should be positive because the transformants were rigorously screened by the selective agent. In fact, negative plants are often detected in T_0_ populations. In this study, two of 11 tillers on plant WL8 were transgene negative (Figure 4A). It is very interesting that the middle two tillers were negative in this plant (Figure 4C). Moreover, the foreign integrating elements in positive plants can be stably inherited by tillers. In summary, Ha3 and WL8 were conjoined plants, not chimeric plants. Mosaic or chimeric plants are normally generated when some tissues are positive and other tissues are negative for the transgenes in a plant. Thereby, the transgenes or mutations in mosaic or chimeric plants cannot stably inherit. We speculated that a negative transformant and a positive transformant are tightly grown together like conjoined babies in humans, and the negative transformant can be survived by the resistance of the positive transformant to the selection pressure in the medium. Conjoined plants were originated from different cells, but the different cells are too close to separate during transformation, so the conjoined plants are generated. Just like a conjoined baby, the conjoined plants are independent individuals although they grow combined. Therefore, this is the reason that positive and negative tillers can be detected in a single plant. The conjoined plants such as WL15 from wheat and BL2 and Ha3 from barley in this study also led to different transgenic events.

When the T_0_ transgenic plant in the genome-editing experiment was shown to be a conjoined plant that contained different independent transgenic events (Figure 4C), the T_0_ transgenic plants should be screened for edited mutations by detecting the individual tillers, and this approach could be too labor-intensive. Thus, mixed leaf samples can be used when screening T_0-_edited plants to avoid missing targeted mutations.

### System for Detecting Mutation in Different Wheat Genomes

Wheat is a complex allopolyploid plant harboring three similar genomes, and the most homoeologous genes have very small sequence differences on the A, B, and D genomes. This fact makes genome editing and the subsequent mutation detection extremely challenging in wheat. Current methods for detecting DNA sequence mutations induced by genome editing include PCR-RE (Shan et al., 2014), PCR/RNP (Liang et al., 2018), the T7EI cleavage assay (Vouillot et al., 2015), next generation sequencing (NGS) (Liu et al., 2019b), high-resolution melting analysis (HRMA) (Dahlem et al., 2012), and fluorescent PCR-capillary gel electrophoresis (Ramlee et al., 2015). Even though each method has its shortcomings, PCR-RE and Sanger sequencing are the most direct, convenient, and widely used methods in many laboratories. Moreover, PCR-RE is the best, efficient, and most cost-effective method when a restriction enzyme site exists in the target sequences. PCR-RE can steadily identify heterozygous mutants, biallelic mutants, and un-mutated WT sequences. Based on our experiences detecting wheat mutations induced by CRISPR/Cas9, a detecting system was suggested as follows: the sgRNA was designed with an incorporated restriction enzyme recognition site. For most genes, a target site with a restriction enzyme site can be found. Mixing leaf samples from different tillers of single plants in the T_0_ generation were collected for DNA extraction and PCR detection. Normally, individual conjoined plants in T_0_ generation might be homozygous in T_1_ generation. Mixing samples can help to avoid missing the edited mutants in T_0_ generation. Generally, different primer pairs specific to the homologs on A, B, and D genomes should be used. However, when the mutation frequency is low and/or a large population needs to be screened, conserved primers that can amplify the target regions from the three homologous genes can be used for the initial screening, and then gene-specific primers can be used for mutation detection. When the mutation plants are heterozygous, the detecting results by PCR-RE will show three binds and the largest fragment which is of the same size to the undigested PCR product can be sequenced; when the mutation plants are biallelic, the PCR-RE results will show only one bind, in which the product can be directly sequenced, and the sequence can be further analyzed at http://skl.scau.edu.cn/dsdecode/; in either way, the PCR-RE product can be ligated into a T-vector and then sequenced. When there is no available restriction enzyme site present in the sgRNA sequence, gene-specific primers can be used to first amplify the target regions, and DNA sequencing is followed to determine whether a mutation is present by examining the overlapping peaks in the sgRNA sequence. Finally, the sequencing results were analyzed at http://skl.scau.edu.cn/dsdecode/, or the PCR product is ligated into a vector to be sequenced.

## Conclusion

In this study, we created barley and wheat mutations for the *Nud* gene by CRISPR/Cas9 and provided materials for studying the functions of the target gene in the two crops. The editing efficiencies for the *Nud* gene in barley and wheat were 57.9 and 51.7%, respectively. The biallelic mutant barley plants showed a naked phenotype. A total of five types of homozygous wheat mutation plants for *TaNud* genes were obtained in the T_1_ generation, especially we identified conjoined plants in which different tillers in a T_0_ individual plant were independent transgenic or genome-editing events in barley and wheat, and different genotypes in different tillers could inherit in the T_1_ generation. Therefore, the transgenic or edited wheat and barley plants need to be detected by mixed leaf samples in the T_0_ generation in case of missing some desired events, which might be a candidate method for detecting edited wheat plants to avoid the loss of possible mutations. The mutants of the *HvNud* gene could not only convert the hulled germplasm into naked barley for dietary purposes but also increase the nutrition value in barley grains.

## Data Availability

The original contributions presented in the study are included in the article/Supplementary Material, further inquiries can be directed to the corresponding authors.

## References

[B1] Abdelrahman M. Al-Sadi A. M. Pour-Aboughadareh A. Burritt D. J. Tran L.-S. P. (2018). Genome Editing Using CRISPR/Cas9-targeted Mutagenesis: An Opportunity for Yield Improvements of Crop Plants Grown under Environmental Stresses. Plant Physiol. Biochem. 131, 31–36. 10.1016/j.plaphy.2018.03.012 29628199

[B2] Aoe S. Ichinose Y. Kohyama N. Komae K. Takahashi A. Abe D. (2017). Effects of High β-glucan Barley on Visceral Fat Obesity in Japanese Individuals: A Randomized, Double-Blind Study. Nutrition 42, 1–6. 10.1016/j.nut.2017.05.002 28870472

[B3] Badhan S. Ball A. S. Mantri N. (2021). First Report of CRISPR/Cas9 Mediated DNA-free Editing of *4CL* and *RVE7* Genes in Chickpea Protoplasts. Int. J. Mol. Sci. 22, 396. 10.3390/ijms22010396 PMC7795094 33401455

[B4] Bartlett J. G. Alves S. C. Smedley M. Snape J. W. Harwood W. A. (2008). High-throughput *Agrobacterium*-Mediated Barley Transformation. Plant Methods 4, 22. 10.1186/1746-4811-4-22 18822125 PMC2562381

[B5] Brooks C. Nekrasov V. Lippman Z. B. Van Eck J. (2014). Efficient Gene Editing in Tomato in the First Generation Using the Clustered Regularly Interspaced Short Palindromic repeats/CRISPR-Associated9 System. Plant Physiol. 166, 1292–1297. 10.1104/pp.114.247577 25225186 PMC4226363

[B6] Chapman J. R. Taylor M. R. G. Boulton S. J. (2012). Playing the End Game: DNA Double-Strand Break Repair Pathway Choice. Mol. Cel 47, 497–510. 10.1016/j.molcel.2012.07.029 22920291

[B7] Dahlem T. J. Hoshijima K. Jurynec M. J. Gunther D. Starker C. G. Locke A. S. (2012). Simple Methods for Generating and Detecting Locus-specific Mutations Induced with TALENs in the Zebrafish Genome. Plos Genet. 8, e1002861. 10.1371/journal.pgen.1002861 22916025 PMC3420959

[B8] Fiaz S. Ahmad S. Noor M. Wang X. Younas A. Riaz A. (2019). Applications of the CRISPR/Cas9 System for Rice Grain Quality Improvement: Perspectives and Opportunities. Int. J. Mol. Sci. 20 (4), 888. 10.3390/ijms20040888 PMC6412304 30791357

[B9] Fiaz S. Wang X. Khan S. A. Ahmar S. Noor M. A. Riaz A. (2021). Novel Plant Breeding Techniques to advance Nitrogen Use Efficiency in rice: A Review. GM Crops Food 12, 627–646. 10.1080/21645698.2021.1921545 34034628 PMC9208628

[B10] Filippo J. S. Sung P. Klein H. (2008). Mechanism of Eukaryotic Homologous Recombination. Annu. Rev. Biochem. 77, 229–257. 10.1146/annurev.biochem.77.061306.125255 18275380

[B11] Gasparis S. Przyborowski M. Kała M. Nadolska-Orczyk A. (2019). Knockout of the HvCKX1 or HvCKX3 Gene in Barley (*Hordeum Vulgare* L.) by RNA-Guided *Cas9* Nuclease Affects the Regulation of Cytokinin Metabolism and Root Morphology. Cells 8, 782. 10.3390/cells8080782 PMC6721474 31357516

[B12] Haas M. Schreiber M. Mascher M. (2019). Domestication and Crop Evolution of Wheat and Barley: Genes, Genomics, and Future Directions. J. Integr. Plant Biol. 61, 204–225. 10.1111/jipb.12737 30414305

[B13] Hall T. (2011). BioEdit: an Important Software for Molecular Biology. GERF Bull. Biosci. 2, 60–61.

[B14] Ishida Y. Tsunashima M. Hiei Y. Komari T. (2015). Wheat (*Triticum aestivum* L.) Transformation Using Immature Embryos. Methods Mol. Biol. 1223, 189–198. 10.1007/978-1-4939-1695-5_15 25300841

[B15] Jiang F. Doudna J. A. (2017). CRISPR-Cas9 Structures and Mechanisms. Annu. Rev. Biophys. 46, 505–529. 10.1146/annurev-biophys-062215-010822 28375731

[B16] Kelliher T. Starr D. Richbourg L. Chintamanani S. Delzer B. Nuccio M. L. (2017). MATRILINEAL, a Sperm-specific Phospholipase, Triggers maize Haploid Induction. Nature 542, 105–109. 10.1038/nature20827 28114299

[B17] Kumar N. Galli M. Ordon J. Stuttmann J. Kogel K.-H. Imani J. (2018). Further Analysis of Barley MORC1 Using a Highly Efficient RNA-Guided Cas9 Gene-Editing System. Plant Biotechnol. J. 16, 1892–1903. 10.1111/pbi.12924 29577542 PMC6181210

[B18] Lawrenson T. Shorinola O. Stacey N. Li C. Østergaard L. Patron N. (2015). Induction of Targeted, Heritable Mutations in Barley and *Brassica oleracea* Using RNA-Guided Cas9 Nuclease. Genome Biol. 16, 258. 10.1186/s13059-015-0826-7 26616834 PMC4663725

[B19] Li J.-F. Norville J. E. Aach J. McCormack M. Zhang D. Bush J. (2013). Multiplex and Homologous Recombination-Mediated Genome Editing in *Arabidopsis* and *Nicotiana Benthamiana* Using Guide RNA and Cas9. Nat. Biotechnol. 31, 688–691. 10.1038/nbt.2654 23929339 PMC4078740

[B20] Liang Z. Chen K. Yan Y. Zhang Y. Gao C. (2018). Genotyping Genome-Edited Mutations in Plants Using CRISPR Ribonucleoprotein Complexes. Plant Biotechnol. J. 16, 2053–2062. 10.1111/pbi.12938 29723918 PMC6230946

[B21] Lieber M. R. (2010). The Mechanism of Double-Strand DNA Break Repair by the Nonhomologous DNA End-Joining Pathway. Annu. Rev. Biochem. 79, 181–211. 10.1146/annurev.biochem.052308.093131 20192759 PMC3079308

[B23] Liu H. Wang K. Jia Z. Gong Q. Ye X. (2019a). Editing *TaMTL* Gene Induces Haploid Plants Efficiently by Optimized Agrobacterium-Mediated CRISPR System in Wheat. J. Exp. Bot. 71, 4. 10.1093/jxb/erz529 PMC7031065 31760434

[B24] Liu Q. Wang C. Jiao X. Zhang H. Song L. Li Y. (2019b). Hi-TOM: a Platform for High-Throughput Tracking of Mutations Induced by CRISPR/Cas Systems. Sci. China Life Sci. 62, 1–7. 10.1007/s11427-018-9402-9 30446870

[B25] Liu C. Zhong Y. Qi X. Chen M. Liu Z. Chen C. (2020a). Extension of the *In Vivo* Haploid Induction System from Diploid maize to Hexaploid Wheat. Plant Biotechnol. J. 18, 316–318. 10.1111/pbi.13218 31344311 PMC6953200

[B26] Liu H. Wang K. Tang H. Gong Q. Du L. Pei X. (2020b). CRISPR/Cas9 Editing of Wheat *TaQ* Genes Alters Spike Morphogenesis and Grain Threshability. J. Genet. Genomics 47, 563–575. 10.1016/j.jgg.2020.08.004 33187879

[B28] Ma X. Zhang Q. Zhu Q. Liu W. Chen Y. Qiu R. (2015). A Robust CRISPR/Cas9 System for Convenient, High-Efficiency Multiplex Genome Editing in Monocot and Dicot Plants. Mol. Plant 8, 1274–1284. 10.1016/j.molp.2015.04.007 25917172

[B29] Mushtaq M. Sakina A. Wani S. H. Shikari A. B. Tripathi P. Zaid A. (2019). Harnessing Genome Editing Techniques to Engineer Disease Resistance in Plants. Front. Plant Sci. 10, 550. 10.3389/fpls.2019.00550 31134108 PMC6514154

[B30] Mushtaq M. Mukhtar S. Sakina A. Dar A. A. Bhat R. Deshmukh R. (2020). Tweaking Genome-Editing Approaches for Virus Interference in Crop Plants. Plant Physiol. Biochem. 147, 242–250. 10.1016/j.plaphy.2019.12.022 31881433

[B31] Mushtaq M. Bhat J. A. Mir Z. A. Sakina A. Ali S. Singh A. K. (2021a). CRISPR/Cas Approach: A New Way of Looking at Plant-Abiotic Interactions. J. Plant Physiol. 224-225, 156–162. 10.1016/j.jplph.2018.04.001 29655033

[B32] Mushtaq M. Ahmad Dar A. Skalicky M. Tyagi A. Bhagat N. Basu U. (2021b). CRISPR-based Genome Editing Tools: Insights into Technological Breakthroughs and Future Challenges. Genes 12, 797. 10.3390/genes12060797 34073848 PMC8225059

[B33] Östman E. Rossi E. Larsson H. Brighenti F. Björck I. (2006). Glucose and Insulin Responses in Healthy Men to Barley Bread with Different Levels of (1→3;1→4)-β-Glucans; Predictions Using Fluidity Measurements of *In Vitro* Enzyme Digests. J. Cereal Sci. 43, 230–235. 10.1016/j.jcs.2005.11.001

[B34] Ramlee M. K. Yan T. Cheung A. M. S. Chuah C. T. H. Li S. (2015). High-throughput Genotyping of CRISPR/Cas9-mediated Mutants Using Fluorescent PCR-Capillary Gel Electrophoresis. Sci. Rep. 5, 15587. 10.1038/srep15587 26498861 PMC4620477

[B47] Shan Q. Wang Y. Li J. Gao C. (2014). Genome Editing in Rice and Wheat Using the CRISPR/Cas System. Nat. Protoc. 9, 2395–2410. 10.1038/nprot.2014.157 25232936

[B35] Taketa S. Awayama T. Amano S. Sakurai Y. Ichii M. (2006). High-resolution Mapping of the *Nud* Locus Controlling the Naked Caryopsis in Barley. Plant Breed. 125, 337–342. 10.1111/j.1439-0523.2006.01207.x

[B36] Taketa S. Amano S. Tsujino Y. Sato T. Saisho D. Kakeda K. (2008). Barley Grain with Adhering Hulls Is Controlled by an ERF Family Transcription Factor Gene Regulating a Lipid Biosynthesis Pathway. Proc. Natl. Acad. Sci. U.S.A. 105, 4062–4067. 10.1073/pnas.0711034105 18316719 PMC2268812

[B37] Tang H. Liu H. Zhou Y. Liu H. Du L. Wang K. (2021). Fertility Recovery of Wheat Male Sterility Controlled by Ms2 Using CRISPR/Cas9. Plant Biotechnol. J. 19, 224–226. 10.1111/pbi.13482 32970905 PMC7868981

[B38] Tiwari U. Cummins E. (2011). Meta-analysis of the Effect of β-glucan Intake on Blood Cholesterol and Glucose Levels. Nutrition 27, 1008–1016. 10.1016/j.nut.2010.11.006 21470820

[B39] Vouillot L. Thélie A. Pollet N. (2015). Comparison of T7E1 and Surveyor Mismatch Cleavage Assays to Detect Mutations Triggered by Engineered Nucleases. G3 (Bethesda) 5, 407–415. 10.1534/g3.114.015834 25566793 PMC4349094

[B40] Wang K. Liu H. Du L. Ye X. (2016). Generation of Marker-free Transgenic Hexaploid Wheat via anAgrobacterium-Mediated Co-transformation Strategy in Commercial Chinese Wheat Varieties. Plant Biotechnol. J. 15, 614–623. 10.1111/pbi.12660 27862820 PMC5399001

[B41] Wang W. Pan Q. He F. Akhunova A. Chao S. Trick H. (2018). Transgenerational CRISPR-Cas9 Activity Facilitates Multiplex Gene Editing in Allopolyploid Wheat. CRISPR J. 1, 65–74. 10.1089/crispr.2017.0010 30627700 PMC6319321

[B42] Wang K. Gong Q. Ye X. (2020). Recent Developments and Applications of Genetic Transformation and Genome Editing Technologies in Wheat. Theor. Appl. Genet. 133, 1603–1622. 10.1007/s00122-019-03464-4 31654081

[B43] Wu J. Chen C. Xian G. Liu D. Lin L. Yin S. (2020). Engineering Herbicide‐resistant Oilseed Rape by CRISPR/Cas9‐mediated Cytosine Base‐editing. Plant Biotechnol. J. 18, 1857–1859. 10.1111/pbi.13368 32096325 PMC7415781

[B44] Yu S. Long H. Deng G. Pan Z. Liang J. Zeng X. (2016). A Single Nucleotide Polymorphism of *Nud* Converts the Caryopsis Type of Barley (*Hordeum Vulgare* L.). Plant Mol. Biol. Rep. 34, 242–248. 10.1007/s11105-015-0911-9

[B45] Zhang Y. Liang Z. Zong Y. Wang Y. Liu J. Chen K. (2016). Efficient and Transgene-free Genome Editing in Wheat through Transient Expression of CRISPR/Cas9 DNA or RNA. Nat. Commun. 7, 12617. 10.1038/ncomms12617 27558837 PMC5007326

[B46] Zhang Y. Li D. Zhang D. Zhao X. Cao X. Dong L. (2018). Analysis of the Functions ofTaGW2homoeologs in Wheat Grain Weight and Protein Content Traits. Plant J. 94, 857–866. 10.1111/tpj.13903 29570880

