# Supplementary material for: Production of Conjoined Transgenic and Edited Barley and Wheat Plants for Nud Genes Using the CRISPR/SpCas9 System
Source: Front Genet. 2022 May 5;13:873850. doi: 10.3389/fgene.2022.873850 (PMC9117629; doi:10.3389/fgene.2022.873850)
Supplement: Supplementary file 1 [file DataSheet1.docx]

**Fig. S1** Detection of *HvNud* edited mutations in different Ha3 tillers in T_1_ generation by PCR-RE. M: DNA marker; +: positive control; -: negative control; 1-4: four different T_1_ plants of Ha3-1; 5-9: five different T_1_ plants of Ha3-2; 10~13: four different T_1_ plants of Ha3-3; 14-18: five different T_1_ plants of Ha3-4; 19-21: three different T_1_ plants of Ha3-5


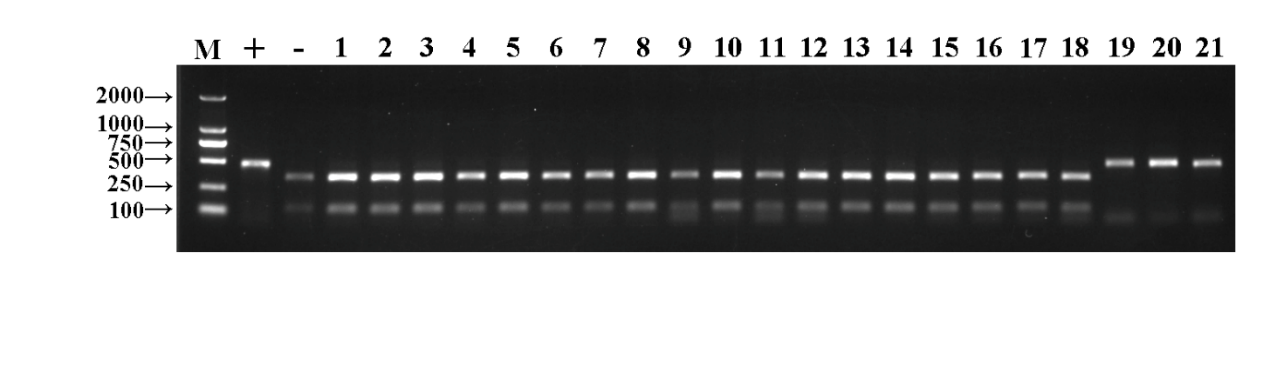


**Fig. S2** Sequence information for the *TaNud* genes located at different chromosomal loci in wheat

**Fig. S3** The map of pWMB110-SpCas9-Nud vector


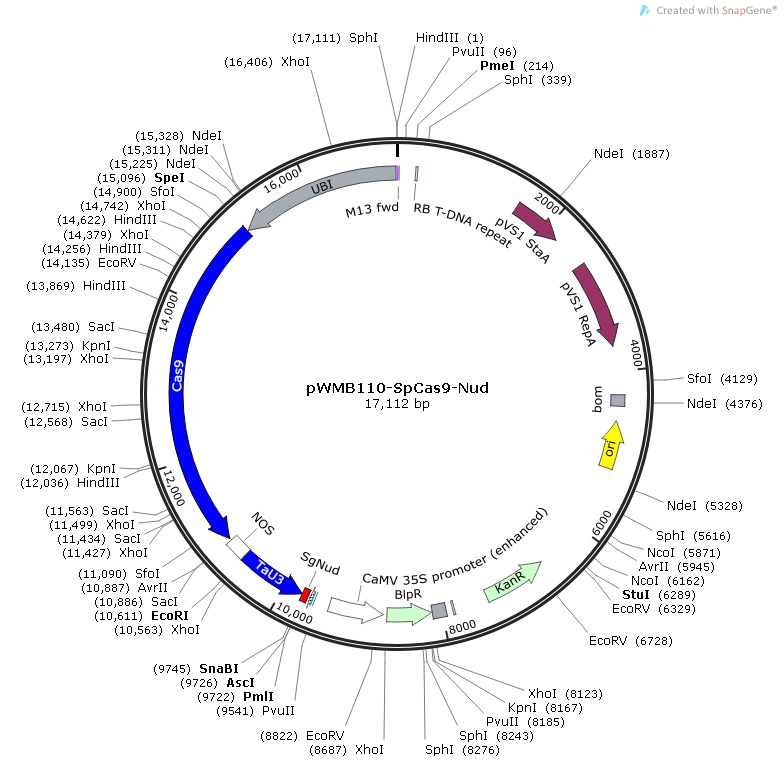


**Table S1** Oligonucleotide primers for PCR amplification of the *Nud* and *SpCas9* genes in wheat and barley

| Primer name | Primer sequence | The size of PCR products |
| --- | --- | --- |
| *HvNud-*330F | GTGGTTGGGCACCTTTGAGACG | 487 |
| *HvNud-*816R | CTCT/CTCGTCCTCCATTGC/TCTCC |  |
| *TaNud-*7AF | GGCAGTGGCGCGGGACGGCCGC | 570 |
| *TaNud-*7AR | TACGACGTGGAGCAATGCAT |  |
| *TaNud-*7BF | TGGTTCCAAATGCATGCATGTATA | 1063 |
| *TaNud-*7BR | TGTGGAGCAGTGATCTTAACTGCT |  |
| *TaNud-*7D-60F | CTTCCGGGCCTCCTCTTCCCTG | 1079 |
| *TaNud-*7D+100R | CAGCTGGTACCTACGATGTGGAGC |  |
| SpCas91052F | TCAAGGCTCTTGTTCGTCAGCA | 1027 |
| SpCas92078R | TTGCCGCTCTGCTTATCCCTGA |  |

**Table S2** Summary of mutations for *HvNud* gene in T_0_ barley plants

| Line | Knockout type (bp) | [Phenotype](javascript:;) |
| --- | --- | --- |
| BL2 | +1 / 0 | - |
| BL6 | -2 | - |
| Ha1 | +1 | - |
| Ha2 | +1 / -8 | naked |
| Ha3 | -4 / -4+8 / 0 | naked/WT |
| Ha4 | -4 /-13+24 | naked |
| Ha5 | -9 /-17 | - |
| Ha6 | -6 / 0 | WT |
| Ha7 | -6 / 0 | WT |
| Ha8 | +1 / 0 | - |
| Ha9 | +1 / 0 | - |

-: no seed; WT: wild-type

**Table** **S3** Potential off-target sites related to the *Nud* gene target sites and PCR primers for their detection in barley and wheat

| Gene ID | | Potential off-target sites | PCR primers for detection of the off-target sites |
| --- | --- | --- | --- |
| *HORVU4Hr1G004590* | TGGCTGGTTGTTGAGCTCGATGG | | OF-1F: CAGTTATCCTGCTCCACC  OF-1R: AACCAGGAGATAAGACTACC |
| *HORVU5Hr1G086520* | CAGCTCCTTGCCGAGCTCGACGG | | OF-2F: ACCACCAGCCTTCCCTAG  OF-2R: TGAACTGCGTGGGAGACC |
| 7D: *248655204-248655221* | GCTCGGCTTGTTGAGCTCGACGG | | OF-7DF: TCCTGACCAGCCAAACGA  OF-7DR: AGCCACAACAGAGGCAAAGT |
| 7B: *578093056-578093073* | ACTAAGCTTGTTGAGCTCGATGG | | OF-7BF: TGAACCAGAGGGCACAGA  OF-7BR: TCGTTTGCTAACTACCCT |

Nucleotide bases shown in red differ from the target sequence in the sgRNA (CGGCTCCTTGTTGAGCTCGA). The 3-base PAM sequences (NGG) are underlined
